# The Z-scores of cardiac indices among healthy children: a systematic review and meta-analysis

**DOI:** 10.1186/s12872-024-04104-6

**Published:** 2024-08-28

**Authors:** Josephat M. Chinawa, Awoere T. Chinawa, Bartholomew F. Chukwu, Igoche D. Peter

**Affiliations:** 1 Department of Paediatrics, College of Medicine, University of Nigeria, Ituku, Ozalla, Enugu Nigeria; 2 Department of Community Medicine, Enugu State University College of Medicine, Enugu State Nigeria; 3 Department of Paediatrics, Univeristy of Nigeria Ituku Ozalla Enugu, Enugu State, Nigeria; 4 Division of Paediatric Cardiology, Limi Children’s Hospital, Abuja, Nigeria

**Keywords:** Meta-analysis, Children, Cardiac structure, Heterogeneity, Z-score

## Abstract

**Background:**

The application of z-scores in normalizing the cardiac size function and structural dimension will be of immense benefit to the clinician, especially in evaluating children with cardiac anomalies. However, heterogeneity in the obtained z- score results is high, thus a subgroup analysis by region (or continent) to assist healthcare practitioners is necessary.

**Objectives:**

The review aimed to ascertain the overall mean z-scores for cardiac structures and function.

**Methods:**

A thorough search of several databases, EMBASE, PubMed/MEDLINE, and Google Scholar was made. Articles published between January 1999 and December 2023 were recruited, of which the last search was done in December 2023. Keywords used in the search were “z-scores”, Children; echocardiography; cardiac structures; cardiac function; and body surface area (BSA)”. We restricted our search to children. Besides, additional relevant articles were manually searched. PRISMA (Preferred Reporting Items for Systematic Reviews and Meta-Analyses) was used to highlight selected studies using a pre-defined search protocol. The *I*^*2*^ statistics were used to ascertain statistical heterogeneity.

**Results:**

Two hundred and forty citations were identified in our search strategy, of which a total of 34 studies were identified. Twenty-four were excluded from the thirty-four studies. A total of 11 studies met our inclusion criteria shown in the PRISMA. Apart from different z scores reading obtained from various countries and regions, some authors focused on few cardiac parameters while others were exhaustive. The mean z-scores of the cardiac structures from various countries/regions range as follows; The range of Z scores obtained by different studies and regions above are as follows; MV;-1.62-0.7 AV: -1.8 -0.5 TV: -2.71 -0.7; PV ; -1.52- (-0.99) MPA; -1-81 -0.8 LPA;-1.07-0.4; RPA;-0.92- 0.1 IVSD; -0.1.77–1.89 LVPWD; -0.12-1.5 LVPWS; -0.1-0.15 LVPWS; 0.03–0.18 LVIDD; -1.13- (-0.98) LVIDS; -0.84-10.3 respectively. The mean z-score from the pooled studies showed mitral valve diameter as -0.24 ± 0.9 and pulmonary valve annuls as -1.10 ± 0.3. The left ventricular end diastolic diameter is -0.93 ± 0.3 while the left ventricular end systolic diameter is -0.05 ± 0.5. The total pooled sample size of the eleven included studies was 9074 and the mean at 95% interval was 824.9 ± 537.344. The pooled mean is presented under the model of the Mean raw (MRAW) column. The heterogeneity discovered among the selected studies was statistically significant.

**Conclusion:**

Due to heterogeneity involved in the reportage of the z-scores of cardiac structures and function, it may be necessary for every region to use their z-scores domiciled in their locale. However, having a pooled mean z-score of cardiac structures and function may be useful in the near future.

## Introduction

The application of z-scores in standardizing the cardiac valve dimension will be of immense benefit to the clinician, especially in evaluating children with cardiac anomalies [1, 2]. Autopsy specimens and angiography were used for several decades in indexing the size of heart valves with body size [3]. However, echocardiography is the easiest way of evaluation of cardiac structures and function indexed to body surface area [1]. For instance, indexing the aortic annular dimension to the surface area could help in the assessment of some vascular anomalies. [4, 5] For instance, aortic root dilation and a defect in the anatomy of aortic architecture are signs of aortic valve disease and surgical intervention. [5] Identifying abnormalities involving the cardiac valve structures and function required accurate knowledge, especially in the application of indexation or normalization of cardiac structures to body surface area [1] For instance, tricuspid and mitral valve sizes are crucial tools that help in determining the etiology and pathophysiology of valvar regurgitation. [6] In addition, valvar dilatation of the tricuspid and mitral valve (MV) could lead to functional and organic regurgitation. [6] Determining the progressive sequel of the dilated cardiac chambers caused by the regurgitation of the valves may help in identifying those valves that needed surgical intervention. [6] Cardiac valve dimension may also be affected by age [7]. Several studies have noted some changes in cardiac valves with age. [7–9] For instance, Gumpangseth et al. [7] in their various studies noted these findings. Cardiac valve parameters, left ventricular assessment, and ascending aorta tend to increase with an increase in age, weight, height, and BSA. [8] The z-score is the only variable that had a strong association with cardiac size when compared to other variables such as height, age, and weight. [10]

The data on studies on estimated z-scores are conflicting. Though there is growing evidence that z-scores remain the standard for estimating cardiac structures and function, and the fact that its application in the treatment of children with heart diseases cannot be overemphasized, yet, growing evidence suggested that several findings on cardiac measures were not consistent. [1, 10] Some findings have suggested some gaps in understanding these novel methods of estimating z-scores. [1, 10] For instance, the estimation of cardiac valve sizes in Nigerian children with heart diseases using the European based z-scores has resulted in the late treatment of such children with attendant mortality and morbidity [10]. There is a likelihood of misinterpretation of reference values of cardiac structures and function from other countries when used in another locale. [11, 12] For instance, Gokhroo et al. [13] noted smaller values on most parameters of the right ventricular dimension of Indian children when compared with those of their western counterparts.Though it may seem advisable for any country to use their locally derived z- scores. However, this my lead to conflicting information and heterogeneity especially when such subject leaves his locale to another zone where such z-score reading may no longer be valid.

This work is therefore aimed at synthesizing the various methods of z-score estimation and harnessing a z-score value via a systematic review and meta-analysis. This may be helpful as it may give a value that can be used across countries and races. Besides, no previous study had estimated the overall z-scores estimation of cardiac structure and function. Hence, having an overall z-score estimation will help to overcome those discrepancies and to have a common understanding of cardiac estimation. The objective of this review was to estimate an overall z-scoring cardiac structure and function in children and to depict heterogeneity.

## Methods

### Study participants

Children aged 1 day to nineteen years were included in the study. Only studies that report outcome data of cardiac structures and function using echocardiography were included, regardless of the echocardiographic views or windows.

### Search strategy

A thorough search of several databases, EMBASE, PubMed/MEDLINE, and Google Scholar was made. Articles published between January 1999 and December 2023 were recruited, of which the last search was done in December 2023. Keywords used in the search were “z-scores”, Children; echocardiography; cardiac structures; cardiac function; and BSA”. We restricted our search to children. Besides, additional relevant articles were manually searched. PRISMA was used to highlight selected studies using a defined search protocols.

### Selection of study

**Fig. 1:**
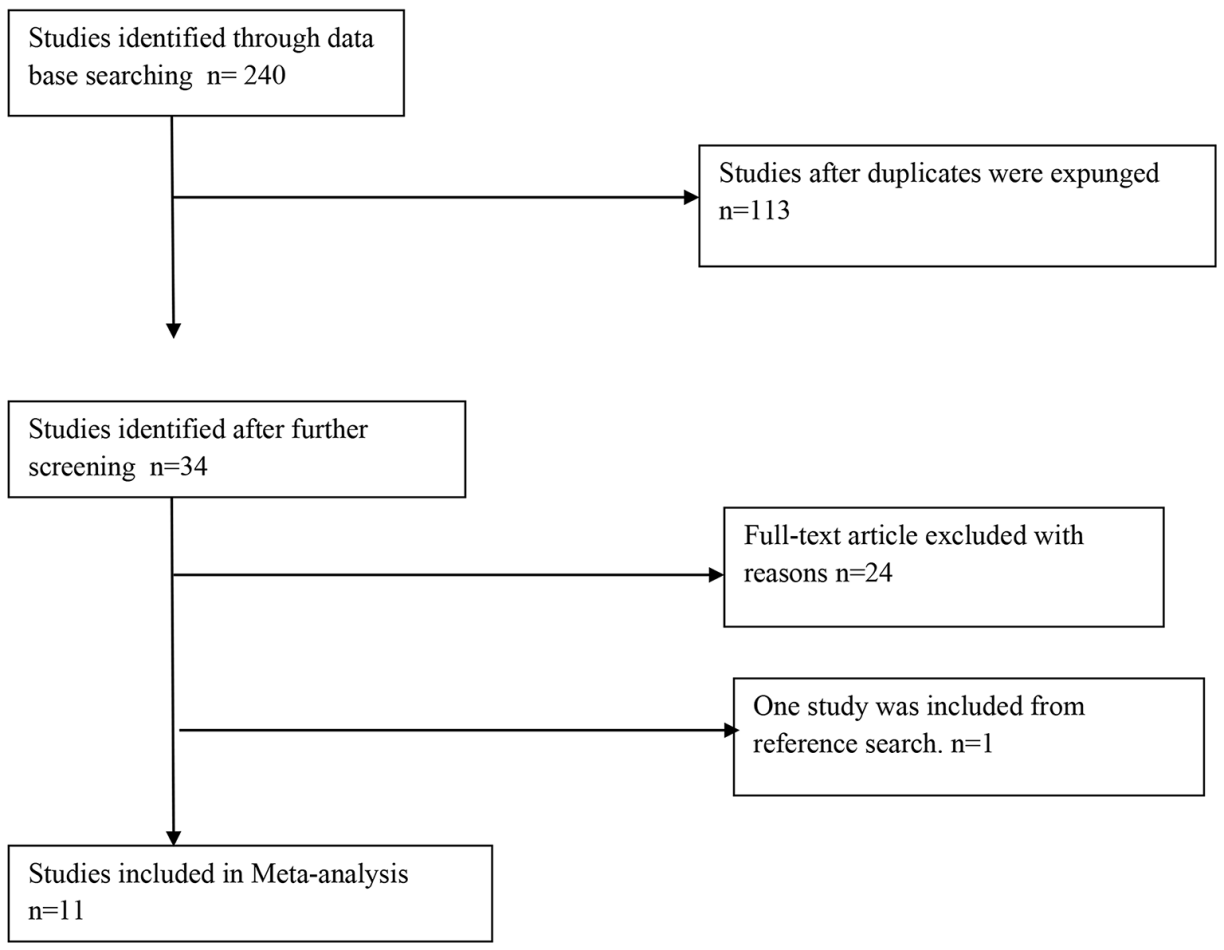
PRIMA flow chart for included studies

All studies that fulfilled the inclusion criteria were selected. This includes: (1) population: Healthy children with normal cardiac structure and function, (2) reported outcome data on z-scores of heart sizes (3) reported outcome data on z-scores of cardiac functions, (4) Age between zero to 19 years. All studies regardless of race were also included. Our exclusion criteria were: Any study with subjects more than 19 years old, case report or case series involving a study population of less than 20 entries, letter to the editor, adult population; z-scores of any population with cardiac anomalies of structure or function; weight-adjusted z-scores of left ventricular M-mode measurements on premature babies, BSA-adjusted z-scores of LV volumes and diameters, data provided as supplementary material to a discussion on ventricular dysfunction following surgery for aortic regurgitation. Duplicated studies were also excluded. A graphical representation of the conceptual framework is also shown in Fig. 1. The literature search was reviewed by 2 reviewers. However, if disagreements between the reviewers ensue, this will be resolved by a meeting with the help of a third independent reviewer. Furthermore, if there was missing data, the reviewers were in touch with the corresponding author. The z-scores of available cardiac structures and function from the authors who fulfilled the inclusion criteria were ascertained. These include the Mitral valve (MV), Aortic valve (AV), Tricuspid valve (TV); Pulmonary valve (PV) ; Main pulmonary artery (MPA); Left pulmonary artery (LPA); Right pulmonary artery (RPA); interventricular septum diameter in diastole (IVSD); left ventricular posterior wall diameter in diastole (LVPWD); left ventricular posterior wall diameter in systole (LVPWS); left ventricular posterior wall diameter in systole (LVPWS); left ventricular internal diameter in diastole (LVIDD); left ventricular internal diameter in systole (LVIDS).

### Data extraction

Socio-demographic data with their z-scores as well as the function of the cardiac structures from all included studies were extracted. Multiple reportage or research work from the same author or study population were not encountered during the meta-analysis. Various z-score characteristics using model regression analysis from different authors were also studied.

### Risk of bias assessment

The studies that met the inclusion criteria were screened and assessed for bias using the modified Newcastle-Ottawa scale for cohort studies [14]. The scale is made up of eight domains, namely: Number of children in the study population (study conducted in a given population = 1 point; multi-center = 0.5 points; single center = 0 points); cohort size (more than one hundred participants = 1 point, between fifty to ninety-nine participants = 0.5 points, fewer than 50 subjects = 0 points); reported information on cardiac structure z-scores (well-defined information = 1 point, information fairly clear but still have some gray areas = 0.5 points, unclear = 0); reported information on cardiac function z-scores (yes = 1 point, no = 0 points); scores of more than six in zero to ten scale are taken as as high-quality, 3–4 as medium quality and < 3 as low-quality, respectively.

### Ethical approval and consent to participate

The approval of the Health Research Ethics Committee of the University of Nigeria Teaching Hospital, Enugu was obtained. In addition, all methods were performed by the relevant guidelines and regulations or declaration of Helsinki.

### Statistical analysis

The pooled z-scores were analyzed with IBM SPSS version 20 software. The pooled z- score of the cardiac structure and function was estimated by the use of the cardiac valve z-score calculator based on the BSA of the pooled subjects. Meta-analysis for means and Forest plots was done using the meta package in R. The *I*^*2*^ statistics was used to ascertain statistical heterogeneity. The higher the percentage of heterogeneity, the more heterogeneous the studies. Heterogeneity is presented in graphs and tables. The pooled mean is presented under the model of the Mean raw (MRAW) column. The higher the percentage of heterogeneity, the more heterogenous the studies. The p-value less than 0.05 indicates that the heterogeneity discovered is statistically significant.

## Results

### Search results and population characteristics

Two hundred and forty citations were identified in our search strategy, of which a total of 34 studies were selected. Twenty-four were excluded from the thirty-four studies due to the reasons below: Preterm babies (*n* = 2), Doppler studies (*n* = 2), studies where isolated structures like left ventricular mass, right heart structures, coronary artery anatomy, tricuspid annular peak systolic velocity (TAPSV), perhaps better known as RV TDI s’, right ventricular systolic function (*n* = 7), syndromic children (*n* = 1) and Adult study (*n* = 2), Chamber volume and area (*n* = 7), bicuspid aortic valve abnormalities (*n* = 3)were ascertained. Finally, a total of 11 studies met our inclusion criteria as shown in the PRISMA flow chart in Fig. 1. See the PRISMA 2020 for Abstracts checklist.

### Characteristics and quality of the studies

**Table 1:** Echocardiographic data of selected study

Author and City where work was done	Year	Age (Y)/BSA	Number of subjects	Diagnostic Method/Study Type/Equations
Daubeney et al. [14] Wessex	1999	0-17.25 0.1–1.9	125	Echocardiography/cross-sectional/ Regression
Kampmann et al. [15] Central Europe	2000	0–18 0–2.0	2036	Digital echocardiography/cross-sectional /Best fitting regression equations
Zilberman et al. [16] Cincinnati	2005	0–18 0–2.0	748	Echocardiography/prospective/z-value nomograms based on BSA
Colan et al. [17] Central America	2006	0–18 0–2.0	68	Echocardiography/Retrospective analysis/multivariate discriminant equation
Overbeek et al. [18] The Netherlands	2006	0–18	700	Echocardiography/Retrospective study/Regression equation
Warren et al. [19] Halifax	2006	0–18 0.25–2.3	317	Echocardiography/Retrospective study/Regression equation
Peterson et al. [20] Michigan	2008	0–18 0–2.0	782	Echocardiography/Prospective study/Regression equation
Gautier et al. [21] Paris	2010	-----	353	Echocardiography/Prospective study/Regression equation
Cantinotti et al. [22] Italy	2014	0–3 0.12–0.67	445	Echocardiography/Prospective/Models with linear, logarithmic, exponential, and square root relationships
Lopez et al. [23] North American population	2017	0–18	3200	Echocardiography/Prospective/ Multivariable regression
Chinawa et al. [24] Nigeria	2023	0–18 0.15–1.90	300	Echocardiography/Prospective/ regression

The various methods of estimating z-scores are elaborated in Table 1 below. Most studies 8 (72.7%) were prospective studies. The z-scores chart was used to derive different z-scores of cardiac structures and function using acceptable BSAs derived from published articles in high impact journal

**Table 2:** z-scores of the various cardiac dimensions in various countries/region

Authors	MV	AV	TV	PV	MPA	LPA	RPA	IVSD	LVPWD	LVPWS	IVSS	LVIDD/LVIDS	
Zilberman et al. (Cincinnati)	0.41	0.52	0.35	−0.99	NA	NA	NA	NA	NA	NA	NA	NA	
Daubeney et al. (Wessex)	−1.54	−1.8	−2.71	−1.53	−0.13	−1.07	−0.48	NA	NA	NA	NA	NA	
Kampmann et al. (Central Europe)	NA	NA	NA	NA	NA	NA	NA	1.89	0.64	0.15	0.18	−0.53/0.21	
Cantinnoti et al. (Italy)	−1.62	−1.16	0.38	−1.32	−1.81	NA	−0.92	−1.77	−0.12	NA	NA	−2.34/−0.84	
Lopez et al. (North American population)	0.28	0.15	0.10	−1.30	−0.03	−0.2	0.12	−0.21	0.85	NA	NA	−0.92/	
Colan et al. (Central America)	−0.34	NA	NA	NA	NA	NA	NA	NA	NA	NA	NA	NA	NA
Overbeek et al. (The Netherlands)	NA	NA	NA	NA	NA	NA	NA	1.92	0.81	NA	NA	−0.98/	
Petterson et al. (Michigan)	−0.44	−0.36	−0.48	−1.15	−0.38		−0.57	NA	0.89	−0.56	−0.03	−1.13/−0.15	
Gautier et al. (Paris)	−0.43	NA	NA	NA	NA	NA	NA	NA	NA	NA	NA	NA	
Warren et al. (Halifax)	−0.3	NA	NA	NA	NA	NA	NA	NA	NA	NA	NA	NA	
Chinawa et al. (Nigeria)	0.7	0.0	0.7	-0.5	0.8	0.4	0.1	1.1	1.5	-0.1	0.2	-0.1/0.3	

Mitral valve (MV), Aortic valve (AV), Tricuspid valve (TV); Pulmonary valve (PV) ; Main pulmonary artery (MPA); Left pulmonary artery (LPA); Right pulmonary artery (RPA); interventricular septum diameter in diastole (IVSD); left ventricular posterior wall diameter in diastole (LVPWD); left ventricular posterior wall diameter in systole (LVPWS); left ventricular posterior wall diameter in systole (LVPWS); left ventricular internal diameter in diastole (LVIDD); left ventricular internal diameter in systole (LVIDS)

Table 2 showed that apart from different z scores reading obtained from the countries and regions shown above, some authors focused on few cardiac parameters while others were exhaustive. The range of Z scores obtained by different studies and regions above are as follows; MV;-1.62-0.7 AV: -1.8 -0.5 TV: -2.71 -0.7; PV ; -1.52- -0.99 MPA; -1-81 -0.8 LPA;-1.07-0.4; RPA;-0.92- 0.1 IVSD; -0.1.77–1.89 LVPWD; -0.12-1.5 LVPWS; -0.1-0.15 LVPWS; 0.03–0.18 LVIDD; -1.13- -0.98 LVIDS; -0.84-10.3 respectively.

**Table 3:** Cross-validation of existing formulae for estimating BSA (m^2^)

Reference	Formula	Year	Bias ± SD (m^2^)	RMSD (m^2^)	*R* ^2^	CCC
Meeh [25]	0.1053⋅Wt^2/3^	1879	0.029 ± 0.073	0.079	0.937	0.943
Du Bois and Du Bois [26]	0.007184⋅Ht^0.725^⋅Wt^0.425^	1916	0.005 ± 0.030	0.030	0.981	0.990
Sendroy and Cecchini [27]	0.0097⋅(Ht + Wt) − 0.545	1954	−0.022 ± 0.035	0.041	0.976	0.982
Gehan and George [28]	0.0235⋅Ht^0.42246^⋅Wt^0.51456^	1970	0.045 ± 0.038	0.059	0.977	0.965
Haycock et al. [29]	0.024265⋅Ht^0.3964^⋅Wt^0.5378^	1978	0.044 ± 0.044	0.062	0.975	0.962
Mosteller [30]	(Height [cm] × Weight [kg]/3600	1987	0.029 ± 0.035	0.045	0.980	0.979
Mattar [31]	(Ht + Wt − 60)/100	1989	−0.001 ± 0.038	0.038	0.976	0.985
Shuter and Aslani [32]	0.00949⋅Ht^0.655^⋅Wt^0.441^	2000	−0.017 ± 0.028	0.033	0.983	0.988
Tikuisis et al. [33]	Female: 0.01474⋅Ht^0.55^⋅Wt^0.47^ Male: 0.01281⋅Ht^0.6^⋅Wt^0.44^	2001	0.013 ± 0.028	0.031	0.982	0.989
Yu et al. [34]	0.00713989⋅Ht^0.7437^⋅Wt^0.404^	2010	0.002 ± 0.030	0.030	0.980	
Schlich et al. [35]	Female:0.000975482·Ht^1.08^·Wt^0.46^ Male: 0.000579479·Ht^1.24^·Wt^0.38^	2010	−0.090 ± 0.046	0.101	0.954	0.895

The BSA used by all the studies above was that of Haycock et al. However, other methods of estimating BSA and the year of study were as shown in Table 3

**Table 4:** Pooled mean z-score values of cardiac valve structures and functions from the pooled studies

	Minimum	Maximum	Mean z-score	Std. Deviation
mitral valve	-1.62	0.70	-0.24	0.9
aortic valve	-1.80	0.15	-0.50	0.6
Tricuspid valve	-2.71	0.70	-0.14	1.2
pulmonary valve annulus	-1.53	− 0.50	-1.10	0.3
main pulmonary artery	-1.81	0.40	-0.39	0.8
right pulmonary artery	− 0.92	0.12	-0.35	0.4
Left pulmonary artery	-1.77	0.80	-0.56	1.1
Interventricular septum diameter in diastole	− 0.53	1.92	1.28	0.9
Left end diastolic diameter	-2.34	− 0.10	-0.93	0.7
left ventricular posterior wall diameter in diastole	0.12	1.50	0.78	0.4
Interventricular septum diameter in systole	− 0.03	0.20	0.11	0.1
Left ventricular posterior wall diameter in systole	− 0.56	0.15	-0.17	0.3
Left ventricular end systolic diameter	− 0.84	0.30	-0.05	0.5

The mean z-score from the pooled studies is as in Table 4 showing mitral valve diameter a -0.24 ± 0.9 and pulmonary valve annulus, as -1.10 ± 0.3. The left ventricular end diastolic diameter is -0.93 ± 0.3 while the left ventricular end systolic diameter is -0.05 ± 0.5. The total pooled sample size of the eleven included studies was 9074 and the mean at 95% interval was 824.9 ±537.344.

### Evaluation of heterogeneity

Heterogeneity was assessed based on the percentages of *I*^2^ for each reported outcome.

The primary studies that were pooled were observational studies.

The graphs and table presented below shows that there is heterogeneity between the studies. The pooled mean is the mean value that corresponds to the random effects model under the Mean raw (MRAW) column. The heterogeneity discovered among the selected studies were statistically significant. See Figs. 2–14.

**Fig. 2:**
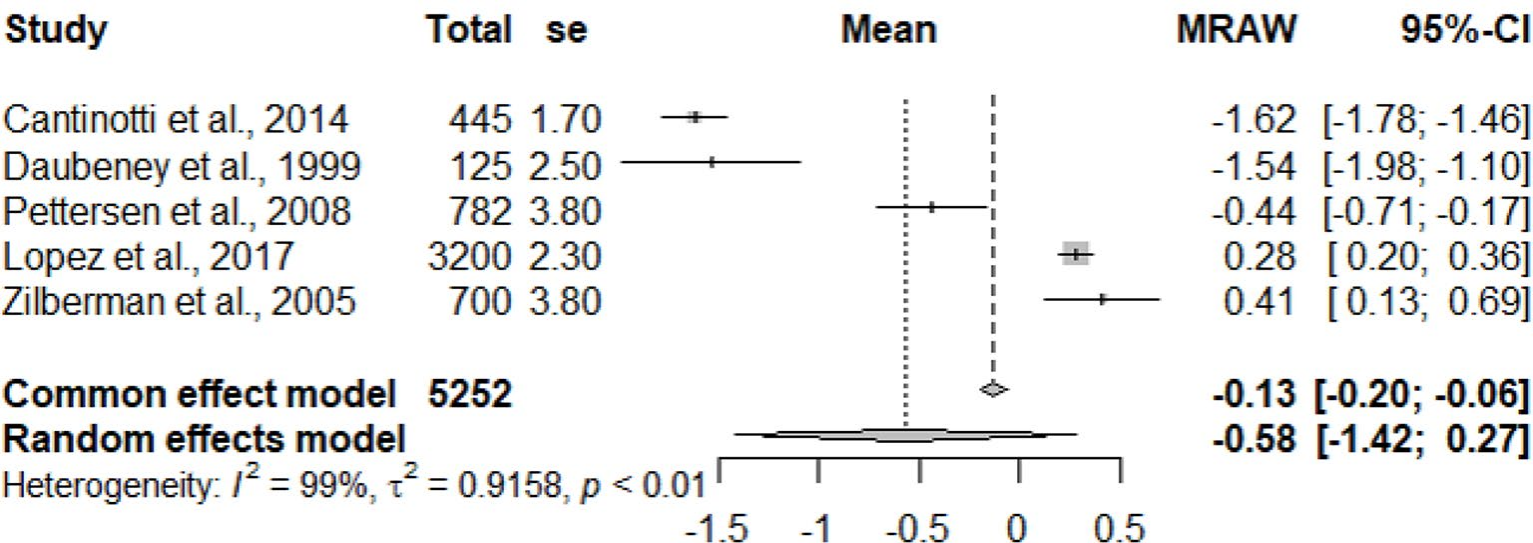
Mitral valve lateral

**Fig. 3:**
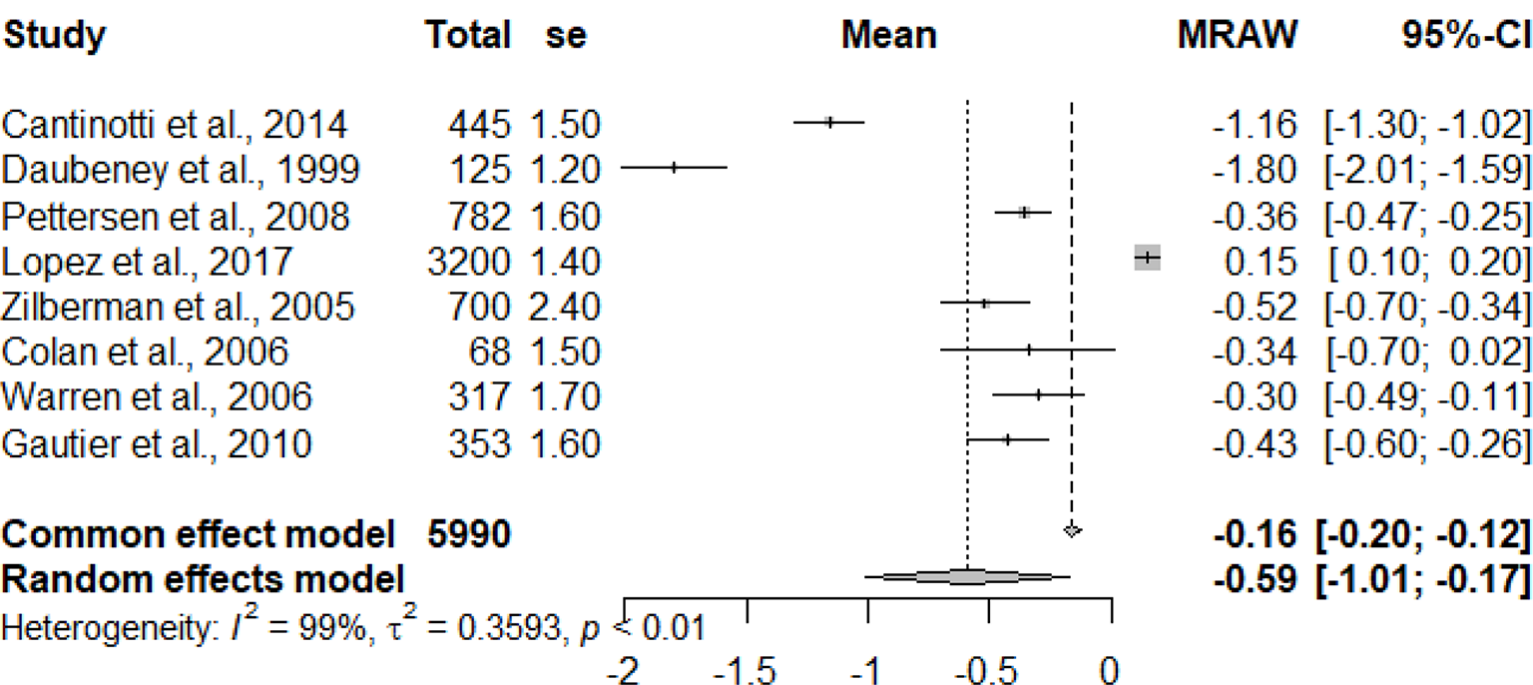
Aortic valve annulus

**Fig. 4:**
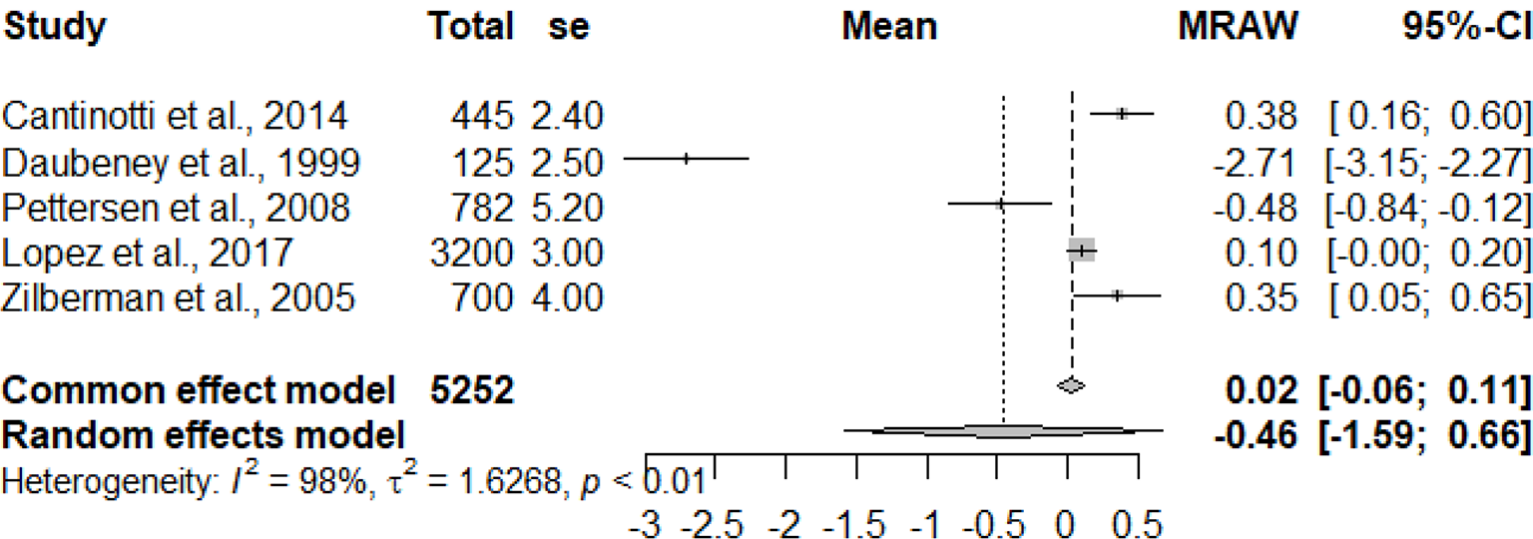
Tricuspid valve lateral

**Fig. 5:**
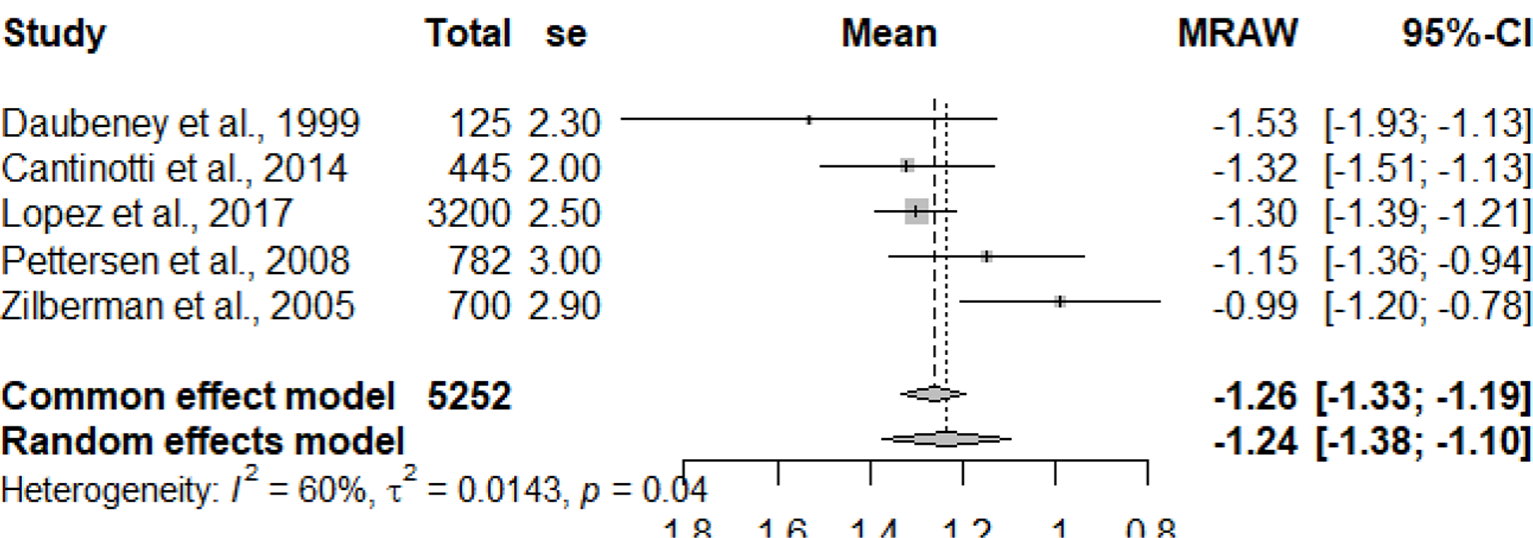
Pulmonary valve annulus

**Fig. 6:**
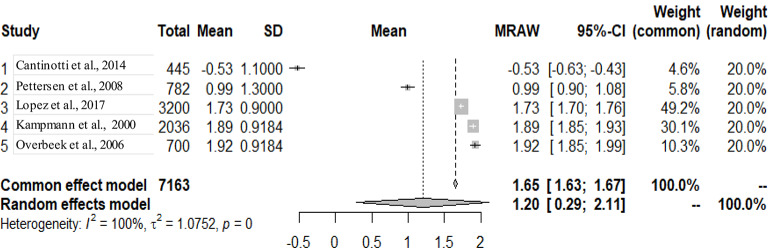
Interventricular septum diastole (IVSD)

**Fig. 7:**
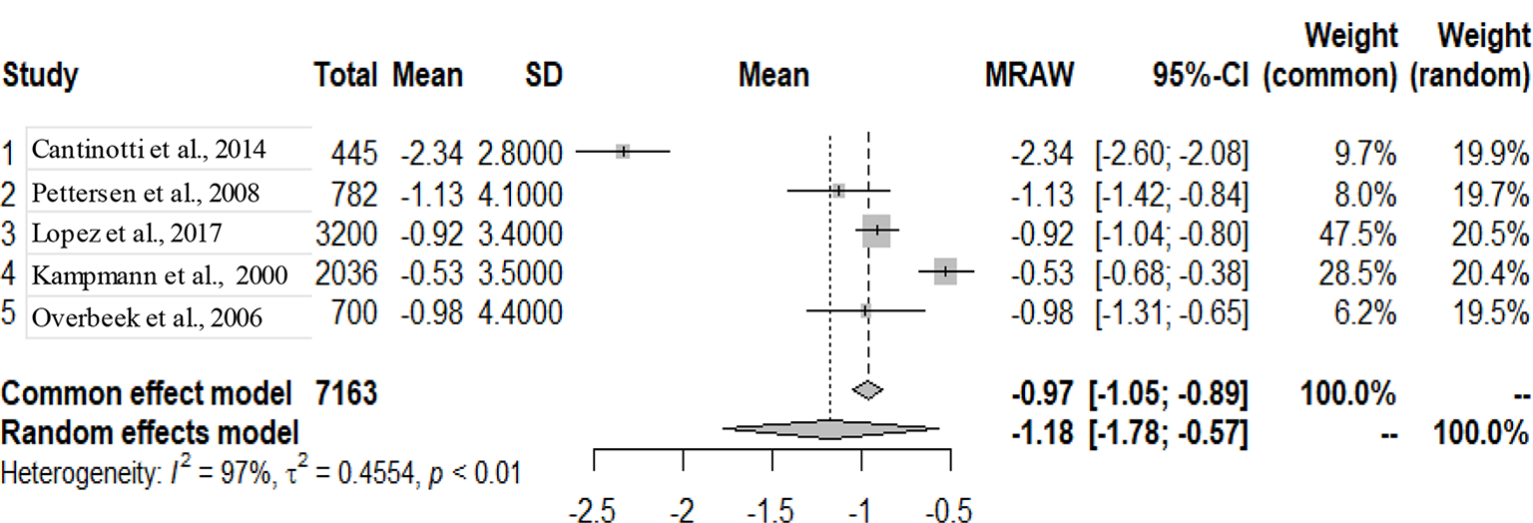
LV end-diastolic diameter

**Fig. 8:**
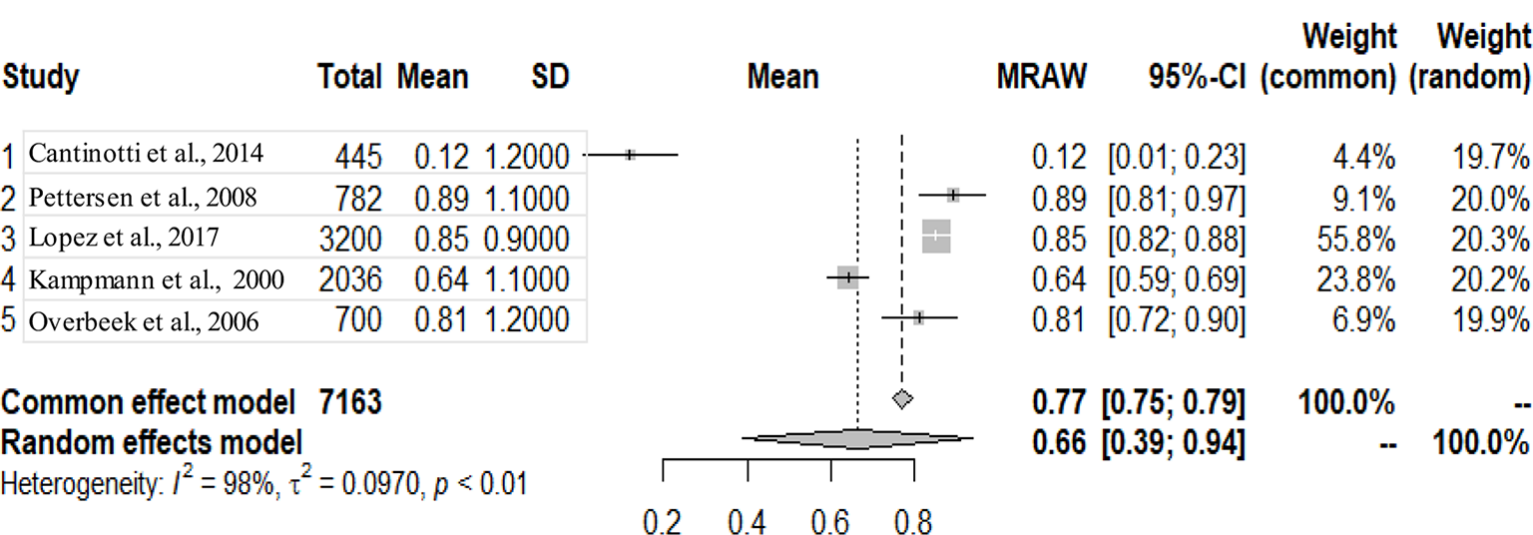
LV posterior wall diastole (LVPWD)

**Fig. 9:**
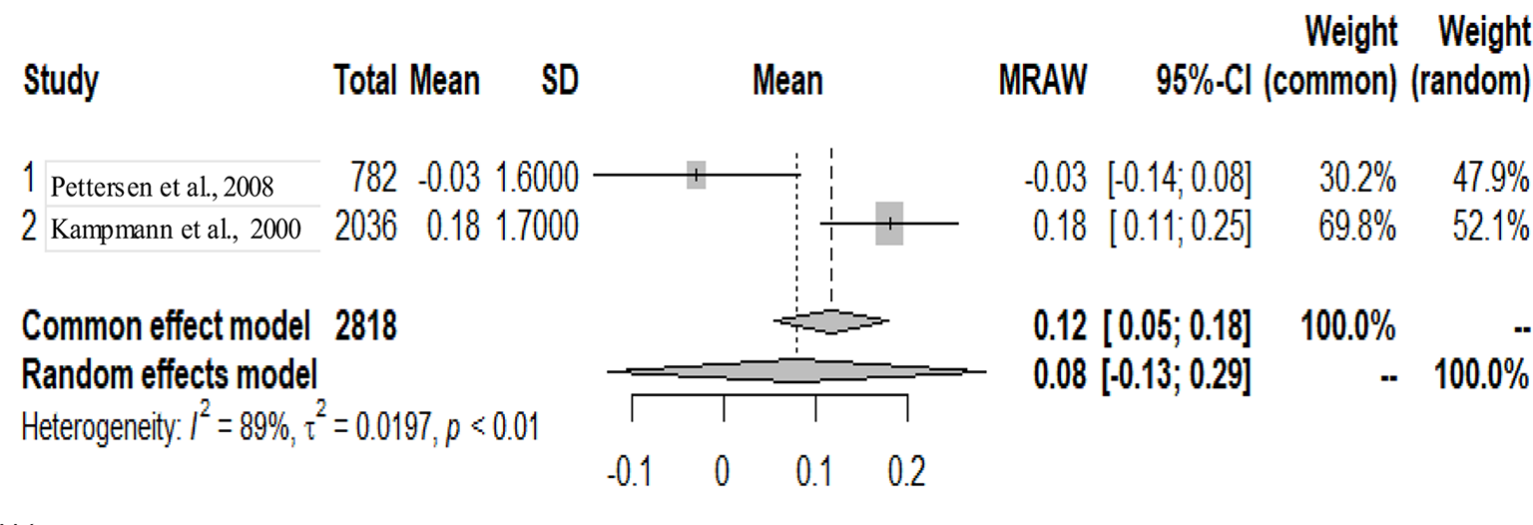
Interventricular septum systole (IVSS)

**Fig. 10:**
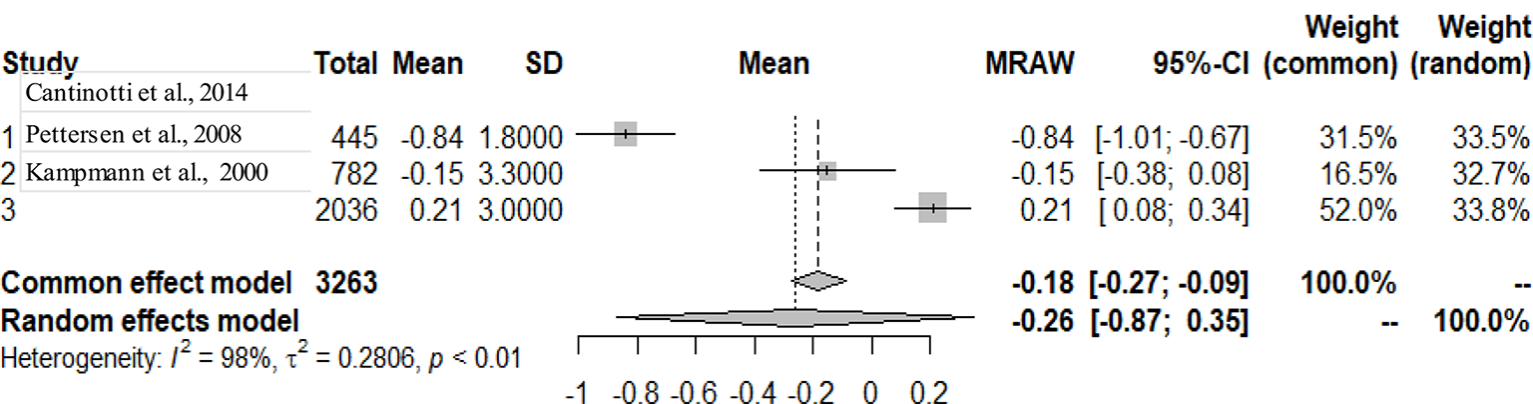
LV end-systolic diameter (LVESD)

**Fig. 11:**
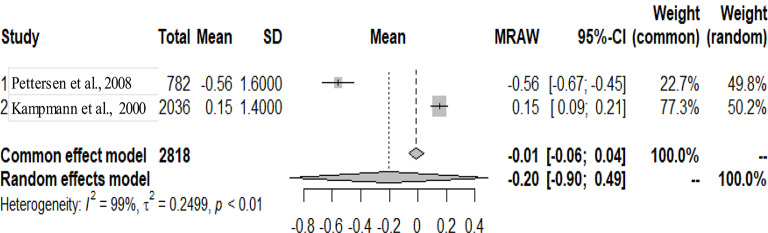
LV posterior wall systole (LVPWS)

**Fig. 12:**
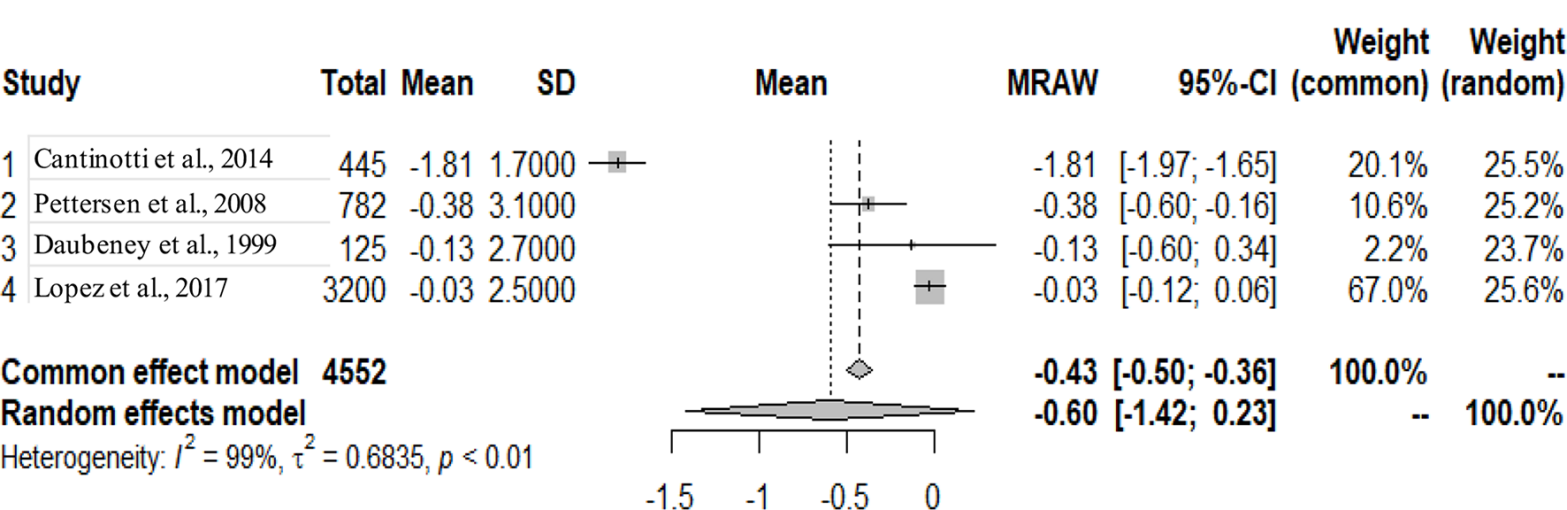
Main pulmonary artery (MPA)

**Fig. 13:**
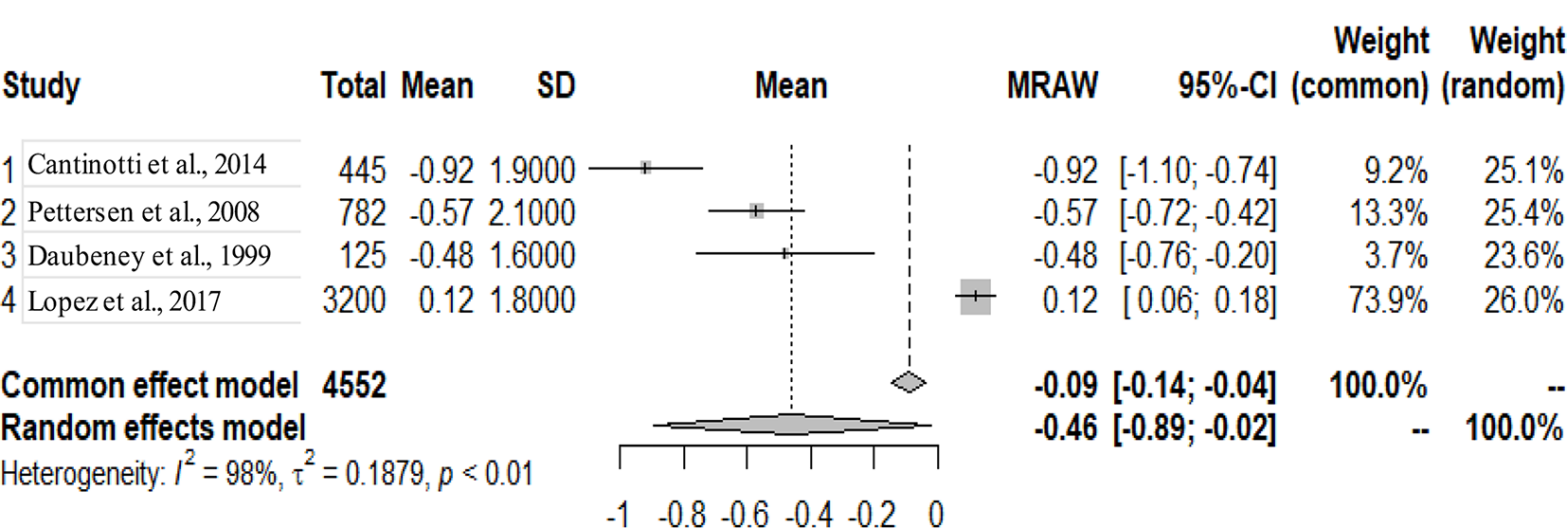
Right pulmonary artery (RPA)

**Fig. 14:**
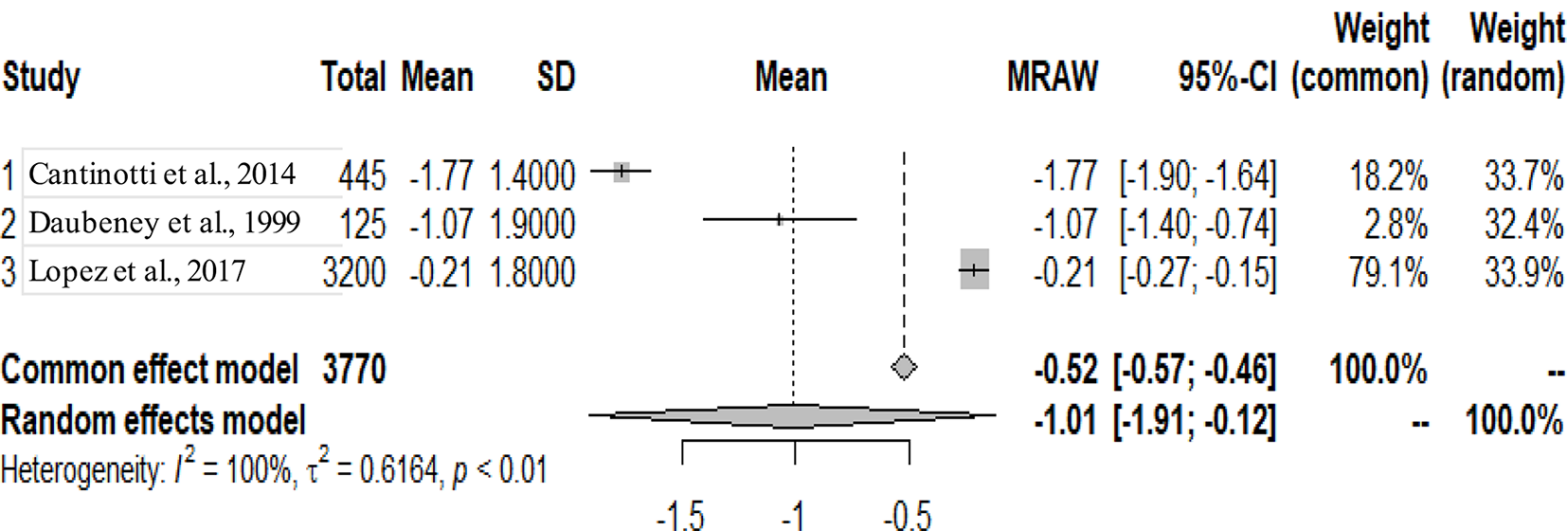
Left pulmonary artery (LPA)

## Discussion

The pooled z-scores showed higher z-score values when compared to some individual studies suggesting heterogeneity among the included studies. The heterogeneity of these studies buttresses the need to standardize the evaluation of the cardiac characteristics among children to ensure that a pool sample reflects the true picture in a study population. Based on our findings, clinicians may reconsider a thorough analysis and indexation of cardiac structures before any medical or surgical procedure especially in open heart surgeries. While it is a generally acceptable fact that z-scores are vital in the approach of a child with heart disease, their overestimation or underestimation may lead to misdiagnosis and late intervention in children in critical care. However, due to limited published literature on the estimation of cardiac z-scores, a pool study using meta-analysis was needed to avert these errors. This work has helped immensely in estimating the z-scores and pattern of measurements of cardiovascular function in healthy children. Several studies on z-scores of cardiac structures and function abound but only a few had their z -scores indexed with weight versus BSA.

Various studies on z-scores for cardiac structures were reviewed in this meta-analysis with their merits and demerits highlighted. For instance, Daubeney et al. [14], using a regression equation, noted the sizes of 15 cardiac structures among 125 normal infants and children. The merit in the above study was that a regression equation was used to delineate BSA with cardiac valve parameters while the work was limited by the fact that cardiac function was not ascertained, and the sample size was not relatively large. Similarly, Kampmann et al. [15] noted measurements of standardized M-mode parameters shown on centile charts where equations that derived the best fit were derived using the 50th centile mark. This work was merited by the fact that a large sample size was used but limited by the use of only M-mode to assess cardiac function. Secondly, other cardiac valves and functions were not ascertained in the study. In the reportage of Zilberman et al. [16], standard deviations and mean values were documented, and the value of z-scores was developed from such values. However, cardiac function was not ascertained. For Colan [17] et al., the z-scores were elicited in a large population of Caucasian neonates, infants, and preschool using “a rigorous statistical design”. The study may be limited by a small sample size. Overbeek [18] et al. also used weight-adjusted z-scores of left ventricular M-mode diameters. Their work was limited by the fact that cardiac valve structures and dimensions were not ascertained. Warren [19] et al. in their findings, used BSA and weight- based-adjusted z-scores of M-mode parameters in estimating the M-Mode among their subjects.Though there is a merit of relatively large sample size and this method is very good in cases of aortic valve surgeries, like Ross procedure, however there is a limitation in the fact that other cardiac valve dimensions and function were not ascertained. Perterson [20] et al. also used a BSA-adjusted z-scores of twenty- one common M-mode and 2D echocardiographic estimation of cardiac structures with an advantage of a large sample size with all cardiac structure and function documented. Gautier [21] et al. documented the dimension of the ascending aorta starting from the aortic root. Though there is a merit of a relatively large sample size and this method is very good in cases of aortic valve surgeries like the Ross procedure, however this work was limited by the paucity of cardiac valve dimensions and function. Cantinotti et al. [22] used a large sample size with the sample frame drawn from Caucasian children in their BSA-adjusted z-scores, derived from using a robust statistical design. Lopez et al. [23] took data from 3200 healthy children who are below eighteen years of age at 19 centers. Chinawa et al. [24] compared their study with the standard Detroit values using regression analysis and noted that BSA (BSA) was a good parameter for independently predicting cardiac valve dimensions.

### Estimation of BSA

There are several formulae for the estimation of BSA [25–35] but the earliest was that of Du Bois and Du Bois in 1916 [25]. The study population in this study was just a sample size of nine patients with eight adults and one child. However, a very small sample size and paucity of children in the study population posed a serious limitation for its usage in clinical medicine. There have been controversies and debates on which parameters will fit the body architecture of the child. [25] However, for most of this estimation, the consensus was to compute the z -scores based on BSA instead of using weight or height alone. There were so many methods used in recent times for the estimation of BSA such as Dubois [26] and Haycock [29], with notable variations in each formula, especially when the weight is low. However, some authors have recommended the use of the Haycock formula, nevertheless, for an accurate comparison, the same formula for BSA should be applied to the one used to estimate the z-scores [28]. 

Although z-scores have major merits in deciphering the pattern of cardiac structures and function in children. However, z-scores are an approximation with some drawbacks. Firstly, the standard deviation and the mean at each body weight vary remarkably between two observers. [36] Secondly, a very large population is vital, especially when a wide range of cardiac sizes is estimated among children. It is therefore pertinent to include a good number of children at extremes of body weight to overcome heteroscedasticity. The errors of using a small sample size may tend to overestimate z-score values for the larger subjects and underestimate z-score values for the smallest children. [36] Finally, errors in measurements may be amplified by the use of z-scores. [37]

BSA is a very crucial index in paediatric practice. [38] for example, the use of BSA is a better index than the use of weight alone for children when normalization of some entities such as glomerular filtration rate, basal metabolic rate, oxygen consumption, and cardiac index is required. Furthermore, BSA has a better correlation with cardiac structures and function than height or weight. [38–42] The application of drug dosing and correction of fluid and electrolyte imbalance are best indexed on BSA than weight, especially when the therapeutic index is low. treatment outcome can be best predicted by the use of BSA. For instance, after coronary artery bypass surgery, mortality becomes very probable among children with a small BSA. [39] However, it is not sacrosanct that the estimation of the BSA is always the best approach. For instance, height is preferred in the estimation of the left ventricular mass [40–43]. Though, for the estimation of BSA, both weight and height are needed factors [42]. However, height is recorded less commonly than weight especially in low-birth-weight children [42]. The use of only weight for the estimation of BSA, where BSA is estimated by the product of a constant and weight indexed to a certain power [42–44] is possible, but the z-score data may be misrepresented. Besides, for some echocardiographic measurements there may be differences in results depending on whether a measurement is made in diastole or systole. [23] Chubb & Simpson [44] in their study demonstrated a relationship between the z-score of mitral valve annulus with values estimated for a 12-month-old male child with height of 75 cm and weight of 10 kg. [40] The algorithms derived by Chubb & Simpson [44] were in tandem with the mean of (z = 0). Nevertheless, there were some notable variations between the different algorithms, particularly at low z-scores.

### Characteristics of various z-scores measurement

Normalization of cardiac structures can be elicited using echocardiographic z-scores based on body size in growing children. [23] There are several studies on the measurement of z-scores of cardiac function and structures in children, for instance, the Paediatric Heart Network (PHN) recently established robust echocardiographic measurements in 3215 healthy children across several races from North America. [23] The most popular models used in Paediatric cardiology practice where regression equations were derived was the PHN and Boston models. Exponential modulation of BSA was harnessed in these models. [45] Another model is the Italian model, this model uses the logarithmic transformations of both BSA, while the Detroit model uses the polynomial transformations of BSA and the logarithmic transformations [21]. Since the z-score schemes are mostly used in both research studies and paediatric clinical practice, it is essential to understand the limitations associated with using more than one model for a particular clinical scenario. A recent study compared the Boston model to the PHN model, Detroit models, and the Italian models and noted a marked difference in the predicted ranges of twelve cardiovascular function and structures [21]. The Detroit model did not factor children with a BSA of more than 2m^2^. Hence, the PHN database whose BSA is greater than 2 m^2^ was not used for subjects in this model [21]. 

### Z-scores and cardiac dimension

A crucial instrument for estimating cardiac size is the use of z-scores [23]. This scoring system uses the mean deviation of a given measurement from the size or age-specific population. [38, 46]

The measurement of cardiac sizes in the paediatric population is best assessed using z- score. The z-scoring index is a standard tool used to assess cardiac structures and function in that it resolves the error of using the standard deviation and mean at each body size point which could cause inter-observer and intra-observer error in data interpretation [46]. Though a degree of inter and intra-observer variability in measurements is difficult to curtail, the z-scores may affect only minimal changes in a large cohort especially at negative z-scores.

To the best of our knowledge, this is the first and the most detailed analysis of the z-scores of cardiac structures that cut across nations. This study showed a detailed and careful search of the literature using set inclusion and exclusion criteria. This study also used various sample frames and populations to reduce the issue of publication bias to the barest minimum.

### Limitation

Our meta-analysis was limited by the heterogeneity in the study population of included studies. Furthermore, meta-analysis did not include aortic-related measurements other than annuls such as STJ or aortic root, ascending aorta. Besides we could not proffer sensitivities of the cardiac valves structural dimension, since we were unable to get the predictive values.

## Conclusion

Due to heterogeneity involved in the reportage of the z-scores of cardiac structures and function, it may be necessary for every region to use their z-scores domiciled in their locale. However, having a pooled mean z-score of cardiac structures and function may be useful in the near future.

## Details of professional editing service

The revised article was edited for English language and syntax by the “Grammarly” English editing service.

## Data Availability

No datasets were generated or analysed during the current study.
